# Paradoxical Tumor Necrosis Factor-Alpha (TNF-α) Inhibitor-Induced Psoriasis: A Systematic Review of Pathogenesis, Clinical Presentation, and Treatment

**DOI:** 10.7759/cureus.42791

**Published:** 2023-08-01

**Authors:** Aditi Chokshi, Michelle Demory Beckler, Anita Laloo, Marc M Kesselman

**Affiliations:** 1 Dermatology, Nova Southeastern University Dr. Kiran C. Patel College of Osteopathic Medicine, Davie, USA; 2 Microbiology and Immunology, Nova Southeastern University Dr. Kiran C. Patel College of Allopathic Medicine, Davie, USA; 3 Rheumatology, Nova Southeastern University Dr. Kiran C. Patel College of Osteopathic Medicine, Davie, USA

**Keywords:** pathogenesis, treatment, paradoxical, tnf-a inhibitor, psoriasis

## Abstract

Tumor necrosis factor-alpha (TNF-α) inhibitors have been shown to be well tolerated among patients with rheumatoid arthritis, inflammatory bowel disease, and psoriasis. Meanwhile, more recently, clinical practice and research efforts have uncovered increasing cases of psoriatic lesion development tied to initiating treatment with a TNF-α inhibitor. The underlying mechanisms associated with this occurrence have yet to be fully elucidated. A review and analysis of cases of paradoxical psoriasis currently published in the literature is warranted. In addition, exploring possible mechanisms of action and potential treatment options associated with favorable outcomes is much needed.

A systematic literature review was performed utilizing PubMed and Google Scholar databases (1992-present), in which 106 cases of paradoxical psoriasis were reviewed. The most common morphology developed was plaque psoriasis vulgaris. There was a female predominance (61.3%), and the most common underlying autoimmune disease was rheumatoid arthritis (45.3%). In addition, the most commonly associated drug with the onset of psoriatic lesions was infliximab (62.3%). Furthermore, the findings suggest that the most well-supported mechanism of action involves the uncontrolled release of interferon-alpha (IFN-α) from plasmacytoid dendritic cells (pDCs) after TNF-α inhibition. While TNF-α inhibitors have been shown to have great benefits to patients with rheumatologic diseases, cases of paradoxical psoriasis demonstrate the importance of close monitoring of patients on TNF-α inhibitors to allow for early recognition, treatment, and potentially change to a different mechanism of action of the medication used to prevent further progression of the inflammatory lesions.

## Introduction and background

**Figure 1:**
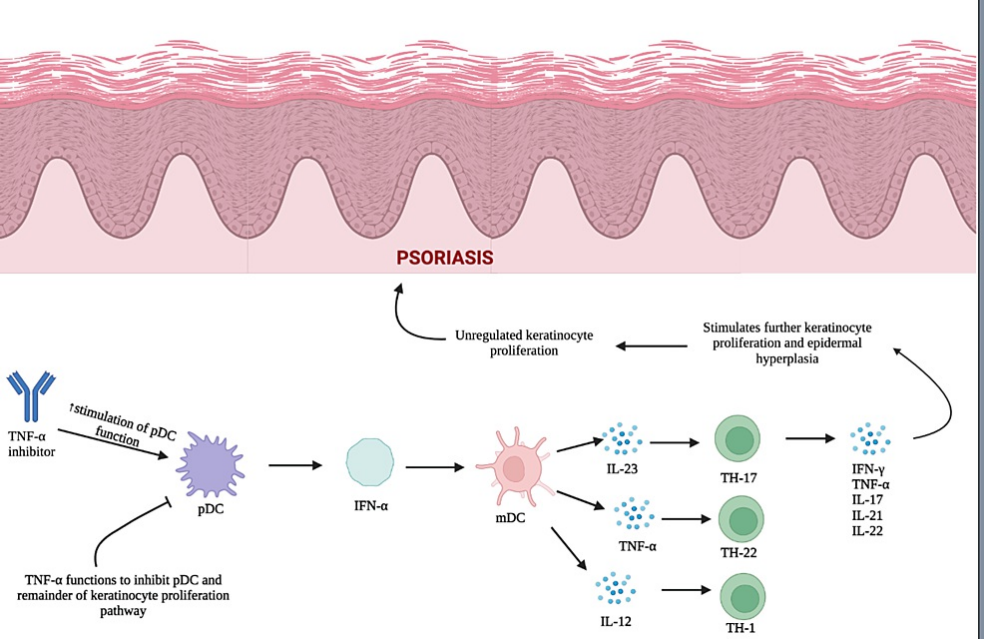
The pathogenesis of psoriasis: This figure illustrates the role of keratinocytes and interferon-alpha (IFN-α) in the pathogenesis of psoriasis. Tumor necrosis factor-alpha (TNF-α) functions to inhibit plasmacytoid dendritic cell (pDC) maturation and the subsequent production of IFN-α. Tumor necrosis factor-alpha inhibition results in unregulated IFN-α production by pDCs and further downstream activation of myeloid dendritic cells (mDCs). This represents the initiation phase of psoriasis, which stimulates the release of inflammatory cytokines and the activation of T-helper cells. Continued inflammatory cytokine production stimulates further keratinocyte proliferation and epidermal hyperplasia and results in the development of psoriatic lesions. [4] Image created with Biorender.com.

Tumor necrosis factor-alpha (TNF-α) is a cytokine generated by activated macrophages, T-lymphocytes, neutrophils, and natural killer (NK) cells to regulate inflammatory responses. Tumor necrosis factor-alpha activates intracellular signaling pathways by binding to either TNF-receptor 1 (TNFR1) or TNF-receptor 2 (TNFR2) [1]. The binding of TNFR1 induces a pro-inflammatory response and apoptosis, while TNFR2 binding triggers anti-inflammatory and cell survival pathways. The balance of TNFR1/TNFR2 signaling helps regulate cell survival, proliferation, differentiation, and death [2,3]. Excess production of TNF-α has been identified as a key factor in the pathogenesis of autoimmune diseases such as rheumatoid arthritis, inflammatory bowel disease, and psoriasis (Figure 1).

Due to the crucial role of TNF-α in the pathogenesis of these diseases, TNF-α inhibitors have revolutionized the treatment of autoimmune diseases, demonstrating increased efficacy compared to alternative treatments. Currently, five TNF-α inhibitors are being used. These drugs include infliximab, adalimumab, certolizumab, pegol, golimumab, and etanercept [1,5]. Although all the TNF-α inhibitors ultimately inhibit the TNF intracellular signaling pathway, they differ in their specific mechanisms of action. Infliximab, adalimumab, and golimumab are bivalent immunoglobulin G (IgG) monoclonal antibodies that competitively inhibit TNF by blocking the interaction of TNFR1 and TNFR2 receptors with TNF. Etanercept is unique as it is a human dimeric fusion protein that binds to TNF with a significantly higher affinity and forms complexes that inhibit TNF from binding to its receptors, further allowing the signaling pathway to continue [6].

Psoriasis is an autoimmune T-cell-mediated disease of the skin characterized by sustained inflammation in the stratum corneum, which stimulates abnormal keratinocyte proliferation and differentiation [7]. A mouse model study demonstrated that the development of psoriatic lesions is dependent on the activation and replication of resident T cells that stimulate epidermal hyperplasia and an angiogenic reaction [8]. It is thought that the proliferation of resident T cells is driven by interferon-alpha/beta (IFN-α/B) production. After skin trauma, infection, or reaction to certain medications, plasmacytoid dendritic cells (pDCs) can infiltrate the skin and secrete IFN-α, which is hypothesized to be the initial step in the development of psoriasis [9,10]. This idea is supported by a 2004 study that found IFN-α production was increased only in the early stages of psoriasis development, yet psoriatic plaques did not show elevated levels of IFN-α [8]. The initial production of Type 1 IFN (IFN-α and IFN-B) then initiates a cascade of cytokine secretion via effector T cells, mainly TH1 and TH17, which results in the production of IFN-γ and interleukin-17 (IL-17), IL-21, and IL-22. Simultaneously, activated myeloid dendritic cells (mDCs) begin to secrete TNF-α, IL-23, and IL-12. Pro-inflammatory cytokines such as TNF-α, IL-17, and IL-23 then function to sustain the maintenance phase of psoriatic inflammation by activating keratinocyte proliferation in the epidermis and the development of plaque-type psoriatic lesions [7].

As the inflammatory response cascades, increased circulating TNF-α results in the infiltration of inflammatory cells from the blood into the skin and dendritic cell activation. TNF-α also increases the activation of keratinocytes, promotes epidermal hyperplasia, and stimulates the nuclear factor kappa B (NF-kB) inflammatory pathway to further increase the production of pro-inflammatory cytokines [11]. A 2004 study using mouse models highlighted the role of TNF-α in the pathogenesis of psoriasis. Pre-psoriatic human skin was engrafted onto 12 mice deficient in Type 1 and 2 interferon receptors (AGR129 mice), which resulted in the development of psoriatic lesions in 90% of the mice. Treatment of mice with TNF-α inhibitors resulted in a significant reduction of T cells in psoriatic lesions. This study strongly supports the role TNF-α plays in the development of psoriatic lesions and that the proliferation of T cells in lesions is dependent on TNF-α production [8]. Due to the central role TNF-α plays in the pathogenesis of psoriasis, TNF-α inhibitors were the first biologic drug approved for the treatment of psoriasis.

However, increasing numbers of cases of paradoxical development of psoriasis in patients with autoimmune diseases treated with TNF-α inhibitors have been reported in the literature. The first case of paradoxical psoriasis induced by TNF-α inhibitors was reported in 2004, but with increasing usage of TNF-α inhibitors, more cases are being reported. The current incidence of paradoxical psoriasis lesions is 2%-5% [12]. Currently, there are more than 100 reports of patients who developed psoriasis after beginning treatment with a TNF-α inhibitor for the management of an alternative autoimmune disease.

Although the number of cases of paradoxical psoriasis continues to increase, there is still no clear mechanism of action established and little research evaluating the treatments that result in the best outcomes for patients who develop these lesions. In this review, we aim to review the current literature regarding proposed mechanisms of action and published cases of TNF-α inhibitor-induced psoriasis. Based on our analysis, we aim to propose treatment options that will help guide clinicians in managing patients to allow for better patient outcomes with complete remission of psoriatic lesions.

Proposed mechanisms of action

Although the mechanism of action tied to the development of psoriatic lesions has yet to be fully elucidated, there are several well-supported theories on the mechanism by which TNF-α inhibitors result in psoriatic lesion development. The most accepted theory is that the TNF-α inhibitor leads to uncontrolled production of IFN-α by pDCs. Plasmacytoid dendritic cells have been found to be increased in the pre-psoriatic skin of psoriasis patients and appear to initiate autoimmune inflammation leading to psoriatic lesion formation. Plasmacytoid dendritic cells infiltrate the skin and produce a surge of IFN-α. As a result, excess levels of IFN-α activate myeloid dendritic cells, which then stimulate pathogenic T cells. This results in the release of the inflammatory cytokines IL-23, TNF-α, and IL-12, which activate T helper cells, stimulate further inflammatory cytokine release and result in unregulated keratinocytes [9]. A study using a human xenograft model of psoriasis demonstrated that blocking IFN-α signaling via treatment with neutralizing antibodies to the IFN-α receptor resulted in the inhibition of both activation and expansion of pathogenic T cells and abrogated the development of psoriatic lesions in mice [9]. Tumor necrosis factor-alpha inhibits both pDC maturation and IFN-α production. Consequently, inhibition of TNF-α can result in unregulated IFN-α production by pDCs and, in turn, the development of psoriatic lesions. When the psoriatic lesion was examined histologically, IFN-α was found to have increased expression in dermal vascular and perivascular lymphocytic infiltrates [13].

In addition to the effect of TNF-α inhibitors on IFN-α and pDCs, it appears that TNF-α inhibitors may also upregulate the production of TH1 and TH17 cells. A 2014 study was the first to show that an increased number of IFN-γ-secreting TH1 and IL-17/IL-22-secreting TH17 cells were found in patients who developed TNF-α inhibitor-induced psoriasis, suggesting a potential link between TNF-α inhibitor treatment and increases in cytokines strongly associated with psoriasis, such as IL-17 and IL-22 [14].

Furthermore, TNF-α inhibitors may also lead to abnormal lymphocyte migration and upregulated expression of CXCR3 ligands, which have been shown to be involved in the development of psoriatic lesions [14]. Meanwhile, few studies have evaluated the correlation between CXCR3 ligands and cases of paradoxical psoriasis.

As opposed to classical psoriasis, it does not appear that auto-reactive T cells are involved in paradoxical psoriasis. In a T-cell-depleted mouse model, treatment with TNF-α inhibitors resulted in paradoxical psoriatic lesion development as a result of an overactive Type 1 IFN-driven inflammatory response. The paradoxical psoriatic lesions had increased dermal accumulation of pDCs, reduced T cell numbers, and higher levels of Type-1 interferon expression when compared with classical psoriatic lesions. [15] This data strongly suggests that paradoxical psoriasis onset is a drug-related side effect, wherein inflammation sustains a positive feedback loop in the innate immune response. Meanwhile, future studies are needed to evaluate new treatment modalities that target pDCs and Type 1 IFN to prevent the development of paradoxical psoriasis [15]. It is also still unclear what triggers the activation of pDCs that leads to the development of paradoxical psoriasis. There has been some suggestion that the presence of IL23R, FBXL19, CTLA4, SLC12A8, and TAP1 polymorphisms may be involved; however, the exact mechanism of action resulting in the development of lesions is still unclear [16].

## Review

Methods

**Figure 2:**
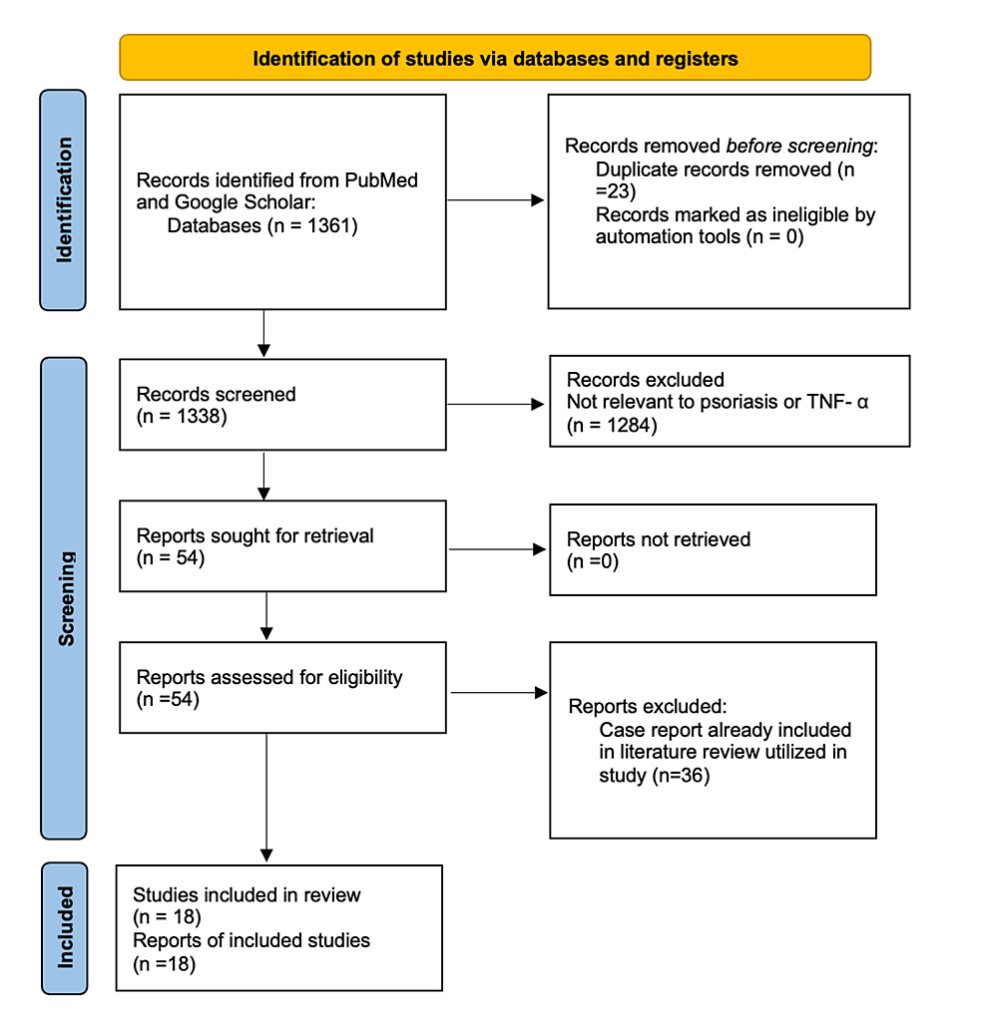
The Preferred Reporting Items for Systemic Reviews and Meta-Analyses (PRISMA) chart of the 18 articles included in this review

A systematic literature review was performed utilizing the PubMed and Google Scholar databases (1992-present). Search terms included "tumor necrosis factor-alpha inhibitor," "TNF-α," and "paradoxical psoriasis" combined with the terms "psoriasis," "pathogenesis," and "treatment." After considering inclusion and exclusion criteria, 18 peer-reviewed publications were identified and utilized (Figure 2).

In addition, we performed an electronic literature search to identify case reports that documented the development of psoriatic lesions after beginning treatment with a TNF-α inhibitor. One hundred and six case reports were included in the final analysis. In each case, the patient's age, the disease being treated, the morphology of psoriasis lesions, and the treatment of lesions combined with the outcome were evaluated and summarized in Table 1.

**Table 1:** Collective data of 106 patients who developed paradoxical psoriasis after TNF-α inhibitor treatment RA: rheumatoid arthritis; UC: ulcerative colitis; CD: Crohn’s disease; AS: ankylosing spondylitis; SPA: spondyloarthritis; SAPHO: synovitis acne hyperostosis osteitis; PsA: psoriatic arthritis; HS: hidradenitis suppurativa; UVB: ultraviolet B

Reference (year)	Primary Disease	Patients Age/Sex	TNF-a Inhibitor	Duration of Treatment	Family/Personal Hx of Psoriasis	Morphology of Lesion	Treatment	Outcome
Jarrett et al., 2003 [17]	RA	60/F	Infliximab	6 weeks	Not Specified	Palmoplantar Pustular Psoriasis	Discontinuation + Oral Prednisolone	Resolution
Thurber et al., 2004 [18]	UC	36/M	Infliximab	24 weeks	No	PS and Palmoplantar PS	Topical Corticosteroids, UV-B	Resolution
Verea et al., 2004 [19]	CD	46/F	Infliximab	6-8 weeks	No	Psoriatic Dermatitis	Discontinuation + Topical Steroids	Resolution
Beuthein et al., 2004 [20]	RA	63/F	Adalimumab	6 weeks	No	Papulopustular Exanthema	Discontinuation	Resolution
Dereure et al., 2004 [21]	RA	47/F	Infliximab	2 months	No	Psoriasis	Topical Corticosteroids + Salicylic Acid	Partial Resolution
	RA	55/F	Infliximab	3 months	No	Psoriasis	Topical corticosteroids + Vitamin D + UV-B	Partial Resolution
Habel et al., 2004 [22]	AS	32/M	Infliximab	8 weeks	No	Psoriasis	Discontinuation + Topical Corticosteroid	Partial Resolution
	AS	27/F	Infliximab	10 months	No	Palmoplantar Psoriasis	Discontinuation + Systemic Corticosteroid	Partial Resolution
	AS	25/M	Etanercept	7 months	Family History	Palmoplantar Psoriasis	Discontinuation + Topicals	Partial Resolution
Sfikakis et al., 2005 [23]	AS	33/F	Infliximab	9 months	No	Psoriasis and Palmoplantar Psoriasis	Switched to Etanercept	Partial Resolution
	RA	65/F	Adalimumab	8 months	No	Psoriasis	Topical Corticosteroids	Resolution
	Behçet's Disease	49/M	Infliximab	6 months	No	Psoriasis and Palmoplantar Psoriasis	Topical Corticosteroids	Slight Improvement
	Behçet's Disease	43/M	Infliximab	6 months	No	Psoriasis and Palmoplantar Psoriasis	Topical Corticosteroids	Resolution
	RA	48/F	Etanercept	6 months	No	Psoriasis and Palmoplantar Psoriasis	Discontinuation + Topical Corticosteroids	Partial Resolution
Grinblat and Scheinberg [24]	RA	37/F	Infliximab	7-9 infusions	No	Psoriasis	Acitretin	Not Tolerated
Michaelson et al., 2005 [25]	RA	62/F	Infliximab	8 weeks	Personal History	Psoriasis	Topical Corticosteroid	Resolution
	RA	50/F	Infliximab	8 weeks	No	Psoriasis	Discontinuation	Resolution
Starmans-Kool et al., 2005 [26]	AS/CD	41/M	Infliximab	4 infusions	Not Specified	Pustular Psoriasis	Topical Clobetasol + Restarted Infliximab Months Later	Resolution
	RA	62/F	Infliximab	5 infusions	Not Specified	Pustular Palmoplantar Psoriasis	Topical Clobetasol + Sulfasalazine + Restart Infliximab 1 Month Later	Resolution
Peramiquel et al., 2005 [27]	CD	29/F	Infliximab	9 infusions	No	Intertriginous PS	Topical Corticosteroid PUVa	Partial Resolution
Zamitski et al., 2005 [28]	RA	50/F	Adalimumab	3 months	Not Specified	Palmoplantar Pustulosis	Topical Corticosteroids	Improvement
Kary et al., 2005 [29]	RA	69/F	Etanercept	1 month	Personal History	Palmoplantar Psoriasis	Discontinuation	Resolution
	RA	65/F	Adalimumab	4 days	No	Pulstulosis Psoriasis Vulgaris	Discontinuation	Resolution
	RA	38/M	Infliximab	3 months	Family History	Psoriasis Vulgaris	Changed to Etanercept	Improvement + Reappearance of Lesions After 6 Weeks
	RA	67/F	Adalimumab	5 months	Family History	Psoriasis Pustulosa	Discontinuation	No Improvement
	RA	49/F	Infliximab	8 months	Personal History	Psoriasis Pustulosa	Topical Corticosteroid	Improvement
	RA	49/F	Etanercept	1 month	Personal History	Psoriasis Vulgaris	Methotrexate Added + Topical Corticosteroid	Not Specified
	RA	63/F	Etanercept	2 months	No	Psoriasis Vulgaris	No Treatment	Stable
	RA	40/F	Adalimumab	11 months	No	Psoriasis Vulgaris	Discontinuation + Cyclosporin Started, Then Switched to Infliximab	No Improvement
Adams et al., 2006 [30]	UC	32/F	Infliximab	2 months	No	Palmoplantar Pustulosis	Topical Corticosteroid	Partial Resolution
	CD	19/M	Infliximab	17 months	No	Palmoplantar Pustulosis	Topical Corticosteroid	Partial Resolution
Matthews et al., 2006 [31]	AS	49/F	Infliximab	8 months	No	Pustular Psoriasis	Increasing Methotrexate Dose	Resolution
	PsA	68/F	Infliximab	Not Specified	Personal History	Intertriginous Psoriasis	Topical Treatment	Resolution
	RA	54/F	Adalimumab	10 months	No	Psoriasis	No Treatment	Resolution
Goiriz et al., 2007 [32]	RA	55/M	Adalimumab	20 months	No	Palmoplantar Psoriasis	Discontinuation + Adalimumab	Significant improvement
	AS	32/M	Infliximab	5 months	No	Plantar Psoriasis	Topical Corticosteroids	Partial Resolution
	PS	52/M	Etanercept	2 months	Personal History	Psoriasis	Topical Corticosteroids	Improvement
	PS	42/F	Etanercept	15 days	Personal History	Psoriasis	Cyclosporine	Remission
	PS	54/F	Etanercept	1 month	Personal History	Psoriasis	Topical Corticosteroids	Improvement
	PS	40/M	Etanercept	14 months	Personal History	Psoriasis	Topical Corticosteroids	Improvement
	PS	58/F	Etanercept	3 months	Personal History	Psoriasis	Topical Corticosteroids	Improvement
	PS	56/M	Etanercept	18 months	Personal History	Psoriasis	Topical Corticosteroids	Improvement
Sari et al., 2006 [33]	RA	30/F	Etanercept	2 months	No	Psoriasis	Discontinuation + Corticosteroid	Resolution
Pirard et al., 2006 [34]	CD	19/F	Infliximab	3 years	Family History	Psoriasis	Topical Corticosteroids	Improved
	RA	47/F	Infliximab	5th infusion	No	Psoriasis	Topical Corticosteroids	Resolution
	AS	29/F	Infliximab	9th infusion	No	Palmoplantar Psoriasis	Topical Corticosteroids	Improved
Volpe et al., 2006 [35]	RA	70/F	Infliximab	9th infusion	No	Psoriasis	Discontinuation + Calcipotriene Ointment	Resolution
	AS	50/M	Infliximab	4 months	No	Psoriasis	Calcipotriene Ointment	Improvement
Gonzalez-Lopez et al., 2006 [36]	CD	39/M	Infliximab	1 month	No	Palmoplantar Psoriasis	Discontinuation + Corticosteroid	Resolution
Aslanidis et al., 2007 [37]	RA	64/F	Adalimumab	18 months	Not Specified	Plaque Psoriasis	Topical Agents	Resolution
	RA	62/F	Adalimumab		Not Specified	Plantar Pustulosis	Topical Agents	Resolution
	AS	24/F	Infliximab	8 months	Not Specified	Palmar Pustulosis	Cyclosporine	Improvement
	RA	29/F	Infliximab	24 months	Not Specified	Plaque Psoriasis	-	Improvement
	SpA	57/M	Infliximab	3 months	Not Specified	Guttate Psoriasis	Topical Agents	Resolution
	RA	76/F	Infliximab	18 months	Not Specified	Plaque Psoriasis	Topical Agents	Resolution
	RA	63/M	Infliximab	14 months	Not Specified	Palmar Pustulosis/Guttate Psoriasis	Cyclosporine	Improvement
	Behcet's Disease	60/M	Infliximab	1.5 months	Not Specified	Palmoplantar Pustulosis	Discontinuation	Resolution
	AS	57/M	Infliximab	3 months	Not Specified	Palmoplantar Pustulosis	Topical Agents/Cyclosporine	Improvement
	RA	47/M	Infliximab	26 months	Not Specified	Plaque Psoriasis	Topical Agents	Improvement
	RA	60/M	Infliximab	4 months	Not Specified	Palmar Pustulosis	-	Stable
	AS	37/M	Infliximab	42 months	Not Specified	Guttate Psoriasis	-	Resolution
Roux et al., 2007 [38]	RA	42/F	Infliximab	30 months	None	Palmoplantar Pustulosis	None	Resolution
	RA	32/F	Infliximab	7 months	None	Palmoplantaris Pustulosis	Discontinuation, Etanercept	Resolution
Severs et al., 2007 [39]	UC	40/F	Infliximab	10 months	Family History	Plaque Psoriasis	Discontinuation + UVB therapy	Improvement
	CD	38/M	Infliximab	42 months	None	Plaque Psoriasis	UVB Therapy	Improvement
	CD	21/M	Infliximab	4 months	None	Pustular Psoriasis	Topical Corticosteroids	Improvement
Ubriana and Van Voorhees e+B16t al., 2007 [40]	RA	65/F	Infliximab	8 weeks	No	Palmoplantar Psoriasis	Discontinuation + Corticosteroid	Resolution After 6 Months
De Gannes et al., 2007 [4]	RA	41/F	Etanercept	26 months	No	Palmoplantar Pustular Psoriasis	Topical Corticosteroids + Topical Calcipotriene	Resolution
	Psoriatic Arthritis	59/F	Infliximab	12 months	Personal History	Psoriasis	Topical Corticosteroids + Topical Calcipotriene	Partial Resolution
	RA	53/F	Etanercept	17 months	Personal History	Psoriasis	Topical Corticosteroids + Topical Calcipotriene	Partial Resolution
	RA	66/F	Etanercept	4 months	No	Psoriasis	Topical Corticosteroids + Topical Calcipotriene	Resolution
	AS	51/M	Etanercept	12 months	No	Psoriasis	Topical Corticosteroids + Topical Calcipotriene	Partial Resolution
	RA	48/F	Etanercept	3 months	No	Pustular Psoriasis	Topical Corticosteroids + Topical Calcipotriene	Partial Resolution
	Juvenile RA	19/F	Adalimumab	3 months	Family History	Palmoplantar Psoriasis	Topical Corticosteroids + Topical Calcipotriene	Partial Resolution
	RA	41/F	Infliximab	2 months	No	Palmoplantar Psoriasis	Topical Corticosteroids + Topical Calcipotriene	Resolution
	RA	52/F	Infliximab	24 months	No	Psoriasis	Topical Corticosteroids + Topical Calcipotriene	Partial resolution
	RA	78/F	Infliximab	2 months	No	Palmoplantar Psoriasis	Topical Corticosteroids + Topical Calcipotriene	Resolution
	RA	57/M	Adalimumab	62 months	No	Psoriasis	Topical Corticosteroids + Topical Calcipotriene	Partial resolution
	RA	50/M	Infliximab	12 months	No	Palmoplantar Psoriasis	Topical Corticosteroids + Topical Calcipotriene	Resolution
	RA	55/F	Adalimumab	36 months	No	Palmoplantar Psoriasis	Topical Corticosteroids + Topical Calcipotriene	Resolution
	RA	49/M	Adalimumab	5 months	No	Pustular Psoriasis	Topical Corticosteroids + Topical Calcipotriene	Resolution
	RA	37/F	Etanercept	24 months	No	Psoriasis	Topical Corticosteroids + Topical Calcipotriene	Partial Resolution
Wollina et al., 2008 [41]	AS	24/F	Infliximab	8 weeks	Not specified	Pustular Psoriasis	Discontinuation + UVB Therapy and Etanercept	Resolution
	CD	22/M	Infliximab	6 weeks	None	Pustular Exanthema	Discontinuation + Prednisolone	Resolution
	Pustular Psoriasis	21/M	Adalimumab	2 weeks	Not specified	Pustular Psoriasis	Discontinuation + Topical Steroids	Resolution
	SAPHO	52/F	Adalimumab	4 weeks	None	Pustular Palmoplantar Psoriasis	Discontinuation + Retinoid Acitretin and PUVA	Resolution
	RA	59/F	Adalimumab	7 months	None	Pustular Psoriasis	Discontinuation + Methotrexate, etanercept, and UVB	Resolution
	AS	52/M	Infliximab	2 years	Family History	Psoriatic Arthritis	Discontinuation + Topical Steroids	Resolution
Manni & Barachini, 2009 [42]	CD	30/F	Infliximab	15 weeks	None	Palmoplantar Pustular Psoriasis	Discontinuation, Corticosteroids, Cyclosporine	Resolution
Nakagomi et al., 2009 [43]	RA	69/F	Infliximab	21 months	Not Specified	Palmoplantar Pustulosis	Discontinuation + Clobetasol and Ciclosporin	Resolution
Bruzzese & Pepe, 2019 [44]	CD	29/M	Infliximab	6 weeks	None	Palmoplantar Pustular Psoriasis	Discontinuation + Corticosteroid	Resolution
Andrew et al., 2010 [45]	Sarcoidosis	58/M	Infliximab	2 years	None	Spongiotic Psoriasiform Dermatitis	Topical Corticosteroids	Improvement
Oh et al., 2010 [46]	AS	53/M	Etanercept	3 years 7 months	Not Specified	Psoriasis	Discontinuation	Improvement
Pyrpasopoulou et al., 2010 [47]	AS	53/M	Infliximab	14 weeks	None	Palmoplantar pustulosis	Cyclosporine + Etanercept	Resolution
Rallis et al., 2010 [48]	PsA	-	Adalimumab	6 months	Not Specified	Palmoplantar Pustular Psoriasis	-	-
Tresh et al., 2012 [49]	Behçet's Disease	55/F	Infliximab	6 weeks	None	Palmoplantar Pustular Psoriasis	Superficial Radiotherapy	Improvement
Kawashima et al., 2013 [50]	CD	22/M	Infliximab	8 weeks	Not Specified	Palmoplantar Pustulosis	Discontinuation + Topical Corticosteroids	Improvement
Broge et al., 2013 [51]	CD	17/F	Infliximab	24 weeks 8 weeks 6 weeks	None	Plaque Psoriasis, Psoriasis Vulgaris, Plaque Psoriasis	Discontinuation + Adalimumab	Resolution
	CD	14/M	Infliximab	8 weeks	None	Psoriasis Vulgaris	Discontinuation + Prednisone and Adalimumab	Resolution
	CD	13/M	Infliximab	6 weeks	None	Plaque Psoriasis	Discontinuation + Topical Corticosteroids. UVB, and methotrexate	Resolution
Peinado, 2015 [52]	CD	32/F	Infliximab	10 years	Not Specified	Scalp Psoriasis	Discontinuation, Corticosteroids, Methotrexate, Vitamin D	Resolution
Gulec et al., 2020 [53]	AS	33/F	Adalimumab	3 months	None	Pustular Psoriasis	Discontinuation, Etanercept, Methotrexate and Cyclosporine	Resolution
	AS	43/F	Infliximab	3 years	None	Palmoplantar Pustular Psoriasis	Topical Corticosteroids	Improvement
	PsA	56/F	Infliximab	7 years	Personal History	PsA	Discontinuation + Secukinumab	Resolution
Irkin et al., 2021 [54]	AS	33/M	Adalimumab	10 years	Not Specified	Palmoplantar, Onycholysis of the Nail, PS of Legs and Back	Discontinuation, Started IL-17 Inhibitor Secukinumab	Significant Regression of Rashes Persistence of Onycholysis
Kanelleas et al., 2022 [55]	HS	4 pts (2M/2F)	Adalimumab	5 months	50% Had a Family History	Plaque Psoriasis	-	-

Results

We identified 40 articles with 106 cases of new-onset TNF-α inhibitor-induced psoriasis (Table 1). Table 2 summarizes the patient demographics.

**Table 2:** A summary of patient demographics

Patients	Total Number (106)
Male	41 (38.6%)
Female	65 (61.3%)
Rheumatoid Arthritis (RA)	48 (45.3%)
Crohn's Disease (CD)	16 (15%)
Ulcerative Colitis (UC)	3 (2.8%)
Ankylosing Spondylitis (AS)	19 (18%)
Hidradenitis Suppurativa	1(0.9%)
Behçet's Disease	4 (3.7%)
Infliximab	66 (62.3%)
Adalimumab	21 (19.8%)
Etanercept	19 (17.9%)
Familial History of Psoriasis	9 (8.4%)
Personal History of Psoriasis	14 (13.2%)
History Not Specified	13 (12.2%)
Resolution After Discontinuation	18 (17%)
Resolution After Discontinuation + Switching TNF-α	10 (9.4%)
Resolution With Continuing Anti-TNF + Corticosteroid	13 (12.2%)
Partial Resolution After Discontinuation and No Steroid	9 (8.5%)
Partial Resolution/Improvement With Continuing Anti-TNF + Corticosteroid	26 (24.5%)

As detailed in Table 2, 61.3% of patients were female and 38.6% were male, with the average age among patients being 45. The rate of developing paradoxical lesions ranged from four days to 10 years after the initiation of TNF-α inhibitors. The most common disease treated that led to the development of paradoxical psoriasis was rheumatoid arthritis (45.3% of patients). Fifteen percent were being treated for Crohn’s disease, 0.03% were being treated for ulcerative colitis, 18% were being treated for ankylosing spondylitis, 0.09% for Behçet's disease, and 0.09% were being treated for hidradenitis suppurativa. Infliximab was the most common TNF-α inhibitor used and resulted in cases of paradoxical psoriasis, totaling 62.3% of the cases reported. Adalimumab was the second most common with 19.8% of cases, followed by etanercept with 17.9% of cases. In addition, 13.2% of patients had a pre-existing personal history of psoriasis, and 0.8% of patients had a family history of psoriasis. The most common morphology developed was plaque psoriasis vulgaris (43%), with the second most common being palmoplantar pustular psoriasis (37.8%). A minority of patients developed two morphologies of psoriasis (6%), with the combination of palmoplantar psoriasis and psoriasis vulgaris being the most common. The most common outcome was partial resolution while continuing treatment with TNF-α inhibitors in conjunction with a topical corticosteroid (24.5%). Furthermore, 17% of patients saw full resolution of lesions after discontinuation of the TNF-α inhibitor or resolution after discontinuation and switching to a different TNF-α inhibitor (9.4%).

Discussion

This review focuses on cases of paradoxical psoriasis provoked by anti-TNF-α drug usage currently published in the literature. While TNF-α inhibitors have been proven to be effective in the majority of psoriasis patients, various cutaneous adverse reactions have been reported, such as erythema, vasculitis, edema, bullous lesions, and lichen planus-like dermatitis [56]. The development of various skin disorders such as psoriasis, lupus-like disorders, eczematiform lesions, and pustular folliculitis has also been reported [57,58]. The development of cutaneous side effects has been reported in several diseases, with autoimmune diseases such as rheumatoid arthritis (45.3%), Crohn’s disease (15%), and ankylosing spondylitis (18%) being the most common among the cases reviewed in this report. Diseases such as ulcerative colitis, Behçet's disease, and juvenile idiopathic arthritis have also been reported as less common causes of cutaneous side effects [41]. The literature suggests smoking, a family history of psoriasis, and the use of immunosuppressive therapies increase the risk of developing cutaneous side effects [59].

There is conflicting evidence regarding the histology of psoriatic lesions. A 2018 study suggested that histologic analysis of paradoxical psoriasis showed high variability among the lesions. Patients exhibited either an eczematiform spongiotic pattern, a psoriasis-like pattern with intraepidermal infiltrates, or a lichenoid pattern. This demonstrates that although some patients with paradoxical psoriasis developed the classic psoriasis histology, others had histopathologic evidence of differing diseases despite appearing to have psoriasis [15]. However, another study revealed that paradoxical psoriatic lesions demonstrate significantly increased levels of mast cells and eosinophils when compared to classical psoriasis lesions histologically. This further supports the theory that paradoxical psoriasis develops due to a different mechanism of action than classic psoriasis [60]. A recent study further supported these findings by showing that paradoxical psoriatic lesions have a marked increase in type-1 IFN expression coupled with a marked dermal accumulation of pDCs when compared with classic psoriasis. TNF-α inhibitors were found to prolong the ability of pDCs to produce type-1 IFN, which drives the differing phenotype of psoriatic lesions in paradoxical psoriasis [15].

In our analysis, the two most common morphologies of paradoxical psoriatic lesions were plaque psoriasis vulgaris and palmoplantar pustulosis (43% and 37.8%, respectively). However, the incidence of palmoplantar pustulosis in the general population with psoriasis is less than 20% [60]. This continues to support the hypothesis that paradoxical psoriasis develops via a different mechanism than classical psoriasis.

While the exact mechanism of action that induces the development of paradoxical psoriasis has yet to be fully elucidated, several well-supported theories continue to be evaluated. The most widely accepted theory implies a key role for pDCs and their production of IFN-α in the induction of psoriatic lesions. Since TNF-α functions to inhibit pDC maturation and IFN-α, TNF-α inhibition may allow for uncontrolled and unregulated production of IFN-α, consequently initiating inflammatory pathways involved in the onset of psoriatic lesions [9]. This idea has been further supported by the discovery of pDCs in early psoriatic lesions and in the skin of patients with autoimmune diseases that are absent in patients with healthy skin. Increased IFN-α expression has also been shown in the dermal vasculature of psoriatic lesions in patients on TNF-α inhibitor treatment [13]. However, further research is still needed to increase our knowledge of the pathogenesis and identify adverse effects.

In the cases reviewed, infliximab (62.3%) was the most reported TNF-α inhibitor to induce psoriatic lesions, with adalimumab (19.8%) being the second most common. This may be attributed to the more common usage of infliximab since it was the first TNF-α inhibitor to receive U.S. Food and Drug Administration (FDA) approval in 1998 for inflammatory bowel disease (IBD) [61]. Whereas adalimumab was approved nine years later, in 2007, for Crohn’s disease [62]. In our analysis, the most common outcome was partial resolution while continuing treatment with TNF-α inhibitors in conjunction with a topical corticosteroid (24.5%). In addition, 17% of patients saw full resolution of lesions after discontinuation of the TNF-α inhibitor (17%) or resolution after discontinuation and switching to a different TNF-α inhibitor (9.4%). However, current literature demonstrates that in patients who chose to switch to a different TNF-α inhibitor, there was an eventual recurrence of lesions in the future [63]. A minority of patients also saw full resolution with the continuation of the TNF-α inhibitor and the addition of a corticosteroid (12.2%). Of those who discontinued treatment with TNF-α inhibitors, only 2% failed to see improvement in lesions. Current literature demonstrates a trend toward better outcomes in patients whose dermatologists initiated multi-modal treatment regimens such as combinations of topical corticosteroids, keratolytics, vitamin D analogs, and ultraviolet (UV)-light therapy [13]. Additionally, potential variables in our analysis of the results included insufficient data on how psoriasis was diagnosed among patients and if patients were on concurrent medications that could be related to the development of psoriatic lesions.

Based on our analysis, we suggest that patients discontinue treatment with their TNF-α inhibitor if another reasonable treatment option for their initial disease is available. Additionally, treatment with topical corticosteroids and phototherapy may be beneficial to those patients who develop mild psoriasis in order to aid in the quicker resolution of lesions. In patients who develop moderate-to-severe psoriasis with significant impacts on quality of life, treatment with methotrexate or systemic therapy allows for an increased probability of resolution. It is important for clinicians to implement close monitoring of patients upon starting treatment with TNF-α inhibitors to allow for early recognition and rapid initiation of treatment for complete resolution of lesions.

## Conclusions

Tumor necrosis factor-alpha (TNF-α) inhibitors have proven to be effective drugs for various rheumatologic diseases, such as rheumatoid arthritis, psoriasis, and inflammatory bowel diseases. While TNF-α inhibitors are generally well tolerated among patients, increasing cases associated with psoriatic lesions after starting TNF-α inhibitor treatment have been reported. Based on our analysis, this sequela is most common in female patients and those using infliximab, which is most commonly associated with the onset of plaque psoriasis, as opposed to other psoriatic morphologies. Topical corticosteroids with discontinuation of TNF-α inhibitors may control or remit the psoriatic lesions. These cases demonstrate the importance of close monitoring of patients on TNF-α inhibitors for early recognition and treatment. Further research may provide a more detailed underlying immunological mechanism of paradoxical psoriasis and help target those individuals’ pre-initiation of TNF-inhibition who may have a predilection to develop paradoxical psoriasis.
